# Abnormal expression of natural mating behaviour of captive adult giant pandas is related to physiological stress

**DOI:** 10.1093/conphys/coae061

**Published:** 2024-09-05

**Authors:** Xueying Wang, Bo Yuan, He Huang, Xiaohui Zhang, Yuliang Liu, Rong Hou, Mingyue Zhang

**Affiliations:** Chengdu Research Base of Giant Panda Breeding, 1375 Panda Road, Northern Suburb, Chengdu, 610081, Sichuan Province, China; Chengdu Research Base of Giant Panda Breeding, 1375 Panda Road, Northern Suburb, Chengdu, 610081, Sichuan Province, China; Chengdu Research Base of Giant Panda Breeding, 1375 Panda Road, Northern Suburb, Chengdu, 610081, Sichuan Province, China; Sichuan Key Laboratory of Conservation Biology for Endangered Wildlife, 1375 Panda Road, Northern Suburb, Chengdu, 610081, SichuanProvince, China; Sichuan Academy of Giant Panda, 1375 Panda Road, Northern Suburb, Chengdu, 610081, Sichuan Province, China; Chengdu Research Base of Giant Panda Breeding, 1375 Panda Road, Northern Suburb, Chengdu, 610081, Sichuan Province, China; Chengdu Research Base of Giant Panda Breeding, 1375 Panda Road, Northern Suburb, Chengdu, 610081, Sichuan Province, China; Sichuan Key Laboratory of Conservation Biology for Endangered Wildlife, 1375 Panda Road, Northern Suburb, Chengdu, 610081, SichuanProvince, China; Sichuan Academy of Giant Panda, 1375 Panda Road, Northern Suburb, Chengdu, 610081, Sichuan Province, China; Chengdu Research Base of Giant Panda Breeding, 1375 Panda Road, Northern Suburb, Chengdu, 610081, Sichuan Province, China; Sichuan Key Laboratory of Conservation Biology for Endangered Wildlife, 1375 Panda Road, Northern Suburb, Chengdu, 610081, SichuanProvince, China; Sichuan Academy of Giant Panda, 1375 Panda Road, Northern Suburb, Chengdu, 610081, Sichuan Province, China; Chengdu Research Base of Giant Panda Breeding, 1375 Panda Road, Northern Suburb, Chengdu, 610081, Sichuan Province, China; Sichuan Key Laboratory of Conservation Biology for Endangered Wildlife, 1375 Panda Road, Northern Suburb, Chengdu, 610081, SichuanProvince, China; Sichuan Academy of Giant Panda, 1375 Panda Road, Northern Suburb, Chengdu, 610081, Sichuan Province, China

**Keywords:** Captive adult giant pandas, cortisol and epinephrine, natural mating behaviour, physiological stress, urine metabolomics

## Abstract

During *ex situ* conservation, the adaptability of giant pandas to environmental changes is greatly challenged. The issue of natural reproduction in captive giant pandas remains unresolved both domestically and internationally. It hypothesized that the restricted natural reproductive capacity may be linked to abnormal mating behavior expression due to physiological stress resulting from incompatible pairings in confined environments. To test this hypothesis, we utilized ultra-high performance liquid chromatographytandem quadrupole-time-of-flight mass spectrometry (UPLC-Q-TOF/MS) to analyse urine metabolites in captive adult giant pandas during their breeding period. Simultaneously, enzyme-linked immunosorbent assay was employed to measure the levels of cortisol and epinephrine in urine, providing insight into the psychological state of captive giant pandas during mate selection by examining all metabolites and related biochemical pathways. This comprehensive approach aims to fully elucidate the physiological mechanisms underlying the decline in natural reproductive capacity. The metabolomics findings indicate that the aberrant expression of natural mating behaviour in captive adult male and female giant pandas may be associated with dysfunction in amino acid metabolic pathways. The activation of these metabolic pathways is linked to psychological stress, such as the tryptophan metabolic pathway and GABAergic synapse pathway. The results of physiological indicators indicate a significant correlation between the expression of natural mating behaviour in captive adult pandas and the hormone urine cortisol, which is associated with physiological stress. These findings indicate that the atypical manifestation of natural mating behaviour in captive adult giant pandas may be associated with physiological stress induced by incompatible pairings within confined environments.

## Teaser

To investigate the challenges of natural reproduction in captive giant pandas, we utilized ultra-high performance liquid chromatography and ELISA to analyze urine metabolites and biochemical indicators. Our results indicate that the unconventional manifestation of mating behavior could be associated with physiological distress resulting from incongruous pairings in restricted habitats.

## Introduction

The giant panda (*Ailuropoda melanoleuca*) is a distinctive species in China and serves as a flagship species for biodiversity conservation (Wei *et al.,* 2015). In order to ensure the preservation of this particular species, China has implemented an *ex situ* conservation and breeding programme. While the implementation of the *ex situ* conservation breeding programme has yielded phased results in giant panda protection, persistent challenges such as the decline of instinctive behaviour within the captive population have significantly impeded the smooth execution of the programme (Xie, 2016; Swaisgood *et al.,* 2018). During *ex situ* conservation, significant alterations have occurred in the habitat of giant pandas (including early weaning in the nursery, maternal separation, non-parental care and exposure to unnatural stressors such as tourist noise), posing considerable challenges to their environmental adaptability. These environmental changes can impact the behaviour of giant pandas, leading to observable distinctions in behavioural patterns between wild and captive pandas, including the manifestation of abnormal behaviours (stereotyped behaviours) and a decline in instinctive behaviours (mating behaviours) (Martin-Wintle *et al.,* 2020; Zhang *et al.,* 2021). Captive giant pandas generally exhibit a low natural reproduction rate. The factors influencing the natural reproductive efficiency of captive giant pandas are complex and variable. The hypothesis suggests that the atypical manifestation of natural mating (NM) behaviour in captive giant pandas may be associated with psychological stress resulting from pair incompatibility within captive environments, given their reputation as a wild species known for strong mate selection (Zhang *et al.,* 2004; Martin-Wintle *et al.,* 2015). However, direct evidence or markers supporting this hypothesis are currently lacking. Our ability to explain the adaptive state of captive giant pandas is currently limited when relying solely on behavioural and hormonal manifestations. Metabolomics is the systematic investigation of the biochemical pathways and interactions of metabolites within biological specimens, providing insight into cellular, tissue, and organismal metabolic activities (Cao *et al.,* 2020). Stress, as a pervasive systemic response, intricately intertwines with the regulation of stress hormones, often heralding substantial alterations in the body’s metabolic landscape. To test our hypothesis, we aim to utilize metabolomics technology to discern subtle differences in metabolite profiles between experimental and control groups. This effort will contribute to a deeper understanding of the underlying biological processes that govern the complex interplay between stress, behaviour and metabolism (Khamis *et al.,* 2017).

In scientific research, stress is commonly categorized into two types based on the intensity and duration of the stressor: acute stress, which typically involves exposure to a single, brief (<30 min) stressor; and chronic stress, which generally entails exposure to a complex, persistent (~1 month) stressor (Chrousos and Gold 1992). Metabolic regulation under stressful conditions serves as a protective mechanism in life processes, with moderate levels of stress aiding the body in adapting to environmental changes and thereby enhancing chances of survival and reproduction. However, excessive or prolonged states of stress, particularly chronic stress, can lead not only to metabolic disorders but also to the onset of severe mental illnesses such as depression (Olff, 1999; Ehlert *et al.,* 2001). The alteration of material metabolism represents a key characteristic of the stress process (Kivimäki *et al.,* 2022). The physiological response elicited by stress typically encompasses a sequence of neuroendocrine activities (Carrasco and Van de Kar 2003). In the context of acute stress, activation of the human body’s sympathetic–adrenal–medullary system and hypothalamic–pituitary–adrenal (HPA) cortex system results in peripheral effects characterized by elevated concentrations of epinephrine and cortisol in the bloodstream. These changes are now relatively well established (McEwen, 2008). Cortisol serves as the principal effector hormone within the HPA axis (HPAA) stress response system and represents a crucial marker for evaluating the intensity of acute stress (O'Connor *et al.,* 2021). When animals experience sudden fright, are relocated to a new environment or undergo long-distance transportation, they exhibit a stress response characterized by a rapid increase in cortisol concentration in the body (Chmelíková *et al.,* 2020; Kovács *et al.,* 2021). Epinephrine, a stress hormone released by the adrenal medulla, plays a pivotal role in acute stress response through its stimulation of the sympathetic nervous system (Gerra *et al.,* 2000). If the stressor persists, it triggers activation of the sympathetic–adrenal medulla system, this results in the release of epinephrine from the adrenal medulla, which is rapidly distributed through the bloodstream to further prepare the body for a response, leading to a variety of physical, psychological and behavioural changes (O'Connor *et al.,* 2021). In addition to alterations in individual biomarkers such as cortisol and epinephrine, metabolomics has also unveiled numerous novel biomarkers that provide insight into the metabolic pathways associated with the pathogenesis of stress-related diseases. For instance, psychosocial stress triggers the activation of certain metabolic pathways that utilize amino acids (Obled *et al.,* 2002), leading to a decrease in the relative concentrations of essential amino acids such as lysine and tryptophan, as well as a reduction in the relative concentration of conditional essential amino acid arginine and vitamin B group nicotinamide (Morgan *et al.,* 2022; Reeds and Jahoor 2001). The impact of stress on the body is more comprehensively reflected through changes in endogenous metabolites of small molecules within organisms.

Hence, in order to investigate the hypothesis that the decline in NM behaviour of captive giant pandas is linked to physiological stress induced by incompatible pairings in confined environments, this study used urine ultra-high-performance liquid chromatography-tandem time-of-flight mass spectrometry (UHPLC-TOF/MS) technique to compare metabolite levels in the urine of captive giant pandas exhibiting different NM behaviours during peak estrus. Additionally, enzyme-linked immunosorbent assay (ELISA) was employed to determine cortisol and epinephrine levels in the urine of male and female captive giant pandas, aiming to elucidate the physiological mechanism underlying the decline in NM behaviour through the analysis of metabolites, related biochemical pathways and hormone levels.

## Materials and Methods

### Animals and ethics statement

In this study, 12 captive giant pandas from the Chengdu Research Base of Giant Panda Breeding (Panda Base) were selected as the subjects for research (Table 1). The essential information and breeding history of the experimental giant pandas are presented in Tables 2 and 3. The pandas were divided into two groups: the NM group, consisting of three female and three male adult giant pandas with successful NM experience (able to produce offspring through NM after reaching adulthood), denoted as F-NM (female-natural mating) and M-NM (male-natural mating). The artificial insemination (AI) group comprised three female and three male adult giant pandas without successful NM experience (unable to produce offspring through NM after reaching adulthood), represented as F-AI (female-artificial insemination) and M-AI (male-artificial insemination). The study protocol was approved by the Institutional Animal Care and Use Committee of Chengdu Research Base of Giant Panda Breeding (approval number: 2020013).

**Table 1:** Information on the giant pandas in this study

Name	Studbook	Groups	Gender	The number of samples collected for metabolomics tests	The number of samples collected for ELISA
Gong Zai	711	NM	Male	2	3
Ying Ying	724	AI	Male	2	3
Cheng Shuang	857	AI	Male	2	3
Mei Lan	649	NM	Male	2	3
Lou Abao	703	NM	Male	2	3
Xi Lan	731	AI	Male	2	3
Er Qiao	823	AI	Female	2	3
Ya Yun	796	AI	Female	2	3
Jing Jing	598	AI	Female	2	3
Mei Lun	870	NM	Female	2	3
Mei Bao	801	NM	Female	2	3
Zhao Mei	990	NM	Female	2	3

Notes: All adult male giant pandas that are unable to mate naturally have attempted NM without success, resulting in the need for semen collection and AI to obtain offspring. In rare instances, adult male giant pandas capable of natural mating may encounter sexual incompatibility, resulting in the need for semen collection and artificial insemination for females. The three selected giant pandas successfully produced offspring through both NM and semen collection, demonstrating their reproductive capabilities.

**Table 2:** Reproductive history information of the captive male giant pandas (The reproductive histories of male giant pandas are informed by statistical data derived from our previously published article in AABS) (Zhang *et al.,* 2021)

Name	Birth year	The number of successful NM (Reproductive history)	The number of offspring produced by NM (Reproductive history)	The number of offspring produced by sperm collection and AI (Reproductive history)	The number of successful NM (2021)	Number of sperm collection (2021)	Sampling time
Ying Ying	2008	0	0	6	0	0	3-24-2021
Cheng Shuang	2012	0	0	1	0	1	2-12-2021
Xi Lan	2008	0	0	2	0	1	3-15-2021
Mei Lan	2006	12	20	4	2	0	3-28-2021
Lou Abao	2007	4	4	2	2	5	3-21-2021
Gong Zai	2008	5	5	2	3	0	3-27-2021

**Notes:** In the 2021 mating season that we sampled, amongst the male NM group, Lou Abao not only successfully completed 2 NM sessions and sired offspring, but also provided semen samples for AI of experimental female giant pandas on five occasions, albeit without successful reproduction. In the male AI group, aside from Cheng Shuang and Xi Lan, who contributed semen once for AI of experimental female giant pandas without producing offspring, Ying Ying did not provide semen samples during that year.

**Table 3:** Reproductive history information of the female giant pandas (The reproductive history of female giant pandas based on historical statistical data from Chengdu Research Base of Giant Panda Breeding for 2000–21)

Name	Birth year	The number of successful NM (Reproductive history)	The number of offspring produced by NM (Reproductive history)	The number of offspring produced by AI (Reproductive history)	Time of the first birth	Sampling time
Er Qiao	2011	0	0	1	2017	3-22-2021
Ya Yun	2010	0	0	1	2018	3-23-2021
Jing Jing	2005	0	0	2	2015	4-15-2021
Mei Lun	2013	1	1	0	2021	3-27-2021
Mei Bao	2010	1	1	1	2019	3-20-2021
Zhao Mei	2010	1	1	0	2021	4-19-2021

**Notes:** In the 2021 mating season, all adult female giant pandas in the female NM group (Mei Lun, Mei Bao and Zhao Mei) successfully engaged in NM for the first time and subsequently gave birth to offspring. Conversely, within the female AI group, none of the adult female giant pandas (Er Qiao, Ya Yun and Jing Jing) successfully completed NM; however, they all successfully produced offspring through AI.

### Feeding and management

All the giant pandas included in this study resided at the Panda Base in Sichuan, China (104°E, 30°N). Each panda was housed individually and provided with consistent indoor and outdoor activity areas, environmental enrichment and surroundings. The feeding, management protocols and food supply were standardized across all individuals.

### Urine sampling

**Figure 1:**
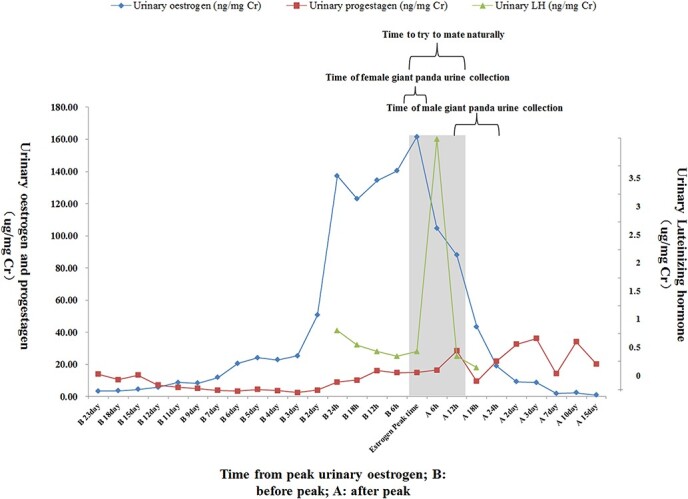
Sampling protocol for adult giant pandas in captivity during the mating season

During this period, all adult male giant pandas were sampled and housed separately in the Moon or Sun delivery room of the Panda Base for the entire breeding season. Adjacent animal enclosures on both sides contained adult female giant pandas in estrus, including those involved in the experiment. Sampling took place after mating attempts during the 2021 mating season (March–April), with urine collected within 3 h following successful NM for Gong Zai, Mei Lan and Lou Abao and within 3 h following unsuccessful NM attempts for Ying Ying, Cheng Shuang and Xi Lan. The non-invasive method was utilized to collect urine samples from male giant pandas during estrus. The Panda Base demonstrated that the appearance of the urinary luteinizing hormone peak indicated the timing of ovulation and facilitated the determination of the optimal time for NM (Cai *et al.,* 2017). We collected urine samples from all female giant pandas during their estrus period. Based on hormone fluctuations, we were able to determine the peak time range of oestrogen (~6 h), and the urine samples required for testing were collected during this specific time frame as illustrated in Fig. 1. After the panda has urinated, it should be lured into a nearby cage and the urine transferred into a 2-ml frozen storage tube using a disposable syringe. Care should be taken to select uncontaminated and undiluted urine, with the date, time and individual name clearly marked. The urine should then be immediately sealed with a sealing film without any chemical treatment, refrigerated, transported back to the laboratory and stored in a −80°C refrigerator for testing.

### Liquid chromatography-mass spectrometry/mass spectrometry analysis

Urine samples were collected in 5-ml vacutainer tubes containing ethylene diamine tetra acetic acid (EDTA) as the chelating agent. The samples were then centrifuged at 1500 × g for 15 min at 4°C. Subsequently, 150-μl aliquots of urine samples were stored at −80°C until UHPLC-Q-TOF/MS analysis. Prior to analysis, the samples were slowly thawed at 4°C and an appropriate quantity was added to a pre-cooled solution of methanol/acetonitrile/water (2:2:1, v/v). The mixture was then vortexed, sonicated at a low temperature for 30 min and allowed to stand at −20°C for 10 min before being centrifuged at 14000 × g for 20 min at 4°C. Following this step, the supernatant was removed and dried under vacuum. Reconstitution of the sample involved adding 100 μl of an aqueous solution of acetonitrile (acetonitrile: water = 1:1, v/v), which was then re-mixed by vortex agitation and centrifuged again at 14 000 × g for 15 min at 4°C prior to supernatant analysis.

The samples were submitted to Shanghai Applied Protein Technology Co., Ltd for untargeted metabolomics analysis, following the detailed detection procedures outlined in our previously published paper (Zhang *et al.,* 2022). The general procedure involves the utilization of the Agilent 1290 Infinity LC ultra-high-performance liquid chromatography system with a HILIC column for sample separation, followed by analysis using the ABTriple TOF 6600 mass spectrometer to collect primary and secondary spectra. The samples were subjected to separation via the Agilent 1290 Infinity LC ultra-high-performance liquid chromatography system and subsequently analysed using the Triple TOF 6600 mass spectrometer (ABSCIEX). Detection was carried out in both positive and negative ion modes of electrospray ionization (ESI). Following data extraction by XCMS software, metabolite structure identification and data preprocessing were conducted prior to experimental data quality assessment, culminating in data analysis.

### ELISA analysis

#### Determination of hormone in urine samples

The concentration of cortisol in urine was quantified using the Cayman Cortisol ELISA Kit (No. 500360) with a sensitivity of 35 pg·ml^−1^, an intra-batch coefficient of variation <13.4% and an inter-batch coefficient of variation <25.8%. The general procedure involved preparing the standard and diluting the sample, followed by adding ELISA buffer, standard, sample, cortisol tracer and cortisol antibody to the enzyme-labelled plate coated with goat anti-mouse immunoglobulin. The plate was then incubated at 4°C overnight. Subsequently, after washing the plates on the following day, Ellman’s reagent and tracer were added and incubated in darkness for 1.5–2 h before measuring absorbance at 405 nm using a Thermo Scientific Multiskan MK3 spectrophotometer.

The concentration of epinephrine in urine was quantified using the IBL Epinephrine ELISA Kit (No. RE59251) with a sensitivity of 0.2 ng·ml^−1^, intra-batch coefficient of variation <8.7%, and inter-batch coefficient of variation <12.1%. Briefly, standard, control and urine samples were added to the boric acid affinity gel-coated macro titration plate. Subsequently, incubation and washing steps were performed with double steaming water, buffer and acylation reagent as required followed by overnight incubation at 4°C. On the following day, the enzyme plate coated with goat anti-rabbit antibodies was utilized for addition of enzyme solution along with standard, control and urine samples. This was followed by sequential addition of epinephrine antiserum, enzyme coupler and PNPP chromogenic solution according to specified incubation and washing steps. The reaction was terminated by adding PNPP termination solution before measuring absorbance at 405 nm using a Thermo Scientific Multiskan MK3 spectrophotometer.

#### Creatinine assays

The urine was diluted 20 times and added to a 96-well plate with 0.05 ml of 0.04 mol·l^−1^ picric acid and 0.05 ml of 0.75 mol·l^−1^ NaOH. After incubation at room temperature (25°C) for 15 min, the optical density (OD) value at 492 nm was measured using a Thermo Scientific Multiskan MK3 spectrophotometer. Samples with a creatinine (Cr) value <0.1 mg·ml^−1^ were considered contaminated with water and excluded from further analysis based on the OD value not being used in these cases.

### Statistical analyses

#### Liquid chromatography-mass spectrometry/mass spectrometry

The data analysis encompassed univariate and multidimensional statistical analysis, differential metabolite screening, correlation analysis of differential metabolite, as well as Kyoto Encyclopedia of Genes and Genomes (KEGG) pathway analysis. Detailed descriptions of the statistical analyses can be found in our previous publications (Zhang *et al.,* 2022).

#### ELISA

Creatinine values were utilized to standardize for variations in water content within each urine sample. The hormone concentration in each urine sample was divided by the creatinine concentration using the following formula: A = creatinine content (mg·ml^−1^) = [creatinine (μg·ml^−1^) × dilution ratio]/1000; B = cortisol content (ng·ml^−1^) = cortisol content per well (ng/well) × dilution release ratio; C = creatinine to correct cortisol (cortisol ng/mg Cr) = B/A. Similar procedures were applied to determine epinephrine levels.

The experimental data were processed using Excel and analysed by SPSS24.0. Group comparisons were conducted using the *t*-test, and the data were presented as x ± SEM. The significance level was set as α = 0.05.

## Results

### Urine metabolomics analysis of giant pandas with different mating behaviours

#### Analysis of intergroup differences in metabolomics experimental data

**Figure 2:**
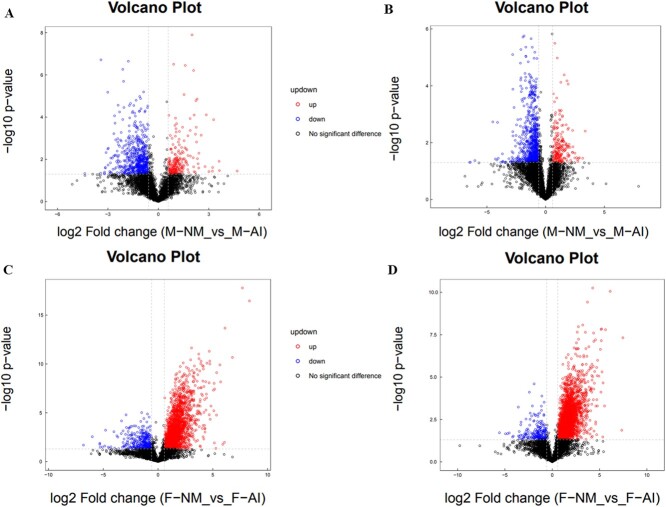
Volcano plot comparing urine metabolites between the NM and AI groups in captive male and female giant pandas; A: captive male giant pandas in positive ion mode; B: captive male giant pandas in negative ion mode; C: captive female giant pandas in positive ion mode; D: captive female giant pandas in negative ion mode. Note: The x-axis in the figure represents the logarithm of log2 Fold Change, whilst the y-axis represents the negative logarithm of -log10 significance *P*-value.

**Figure 3:**
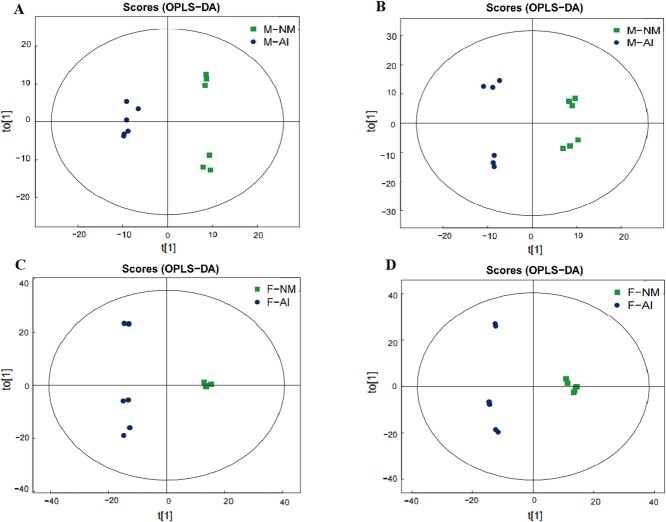
OPLS-DA of urine metabolites between NM group and AI group in captive male and female giant pandas; A: captive male giant pandas in positive ion mode; B: captive male giant pandas in negative ion mode; C: captive male giant pandas in positive ion mode; D: captive male giant pandas in negative ion mode.

**Figure 4:**
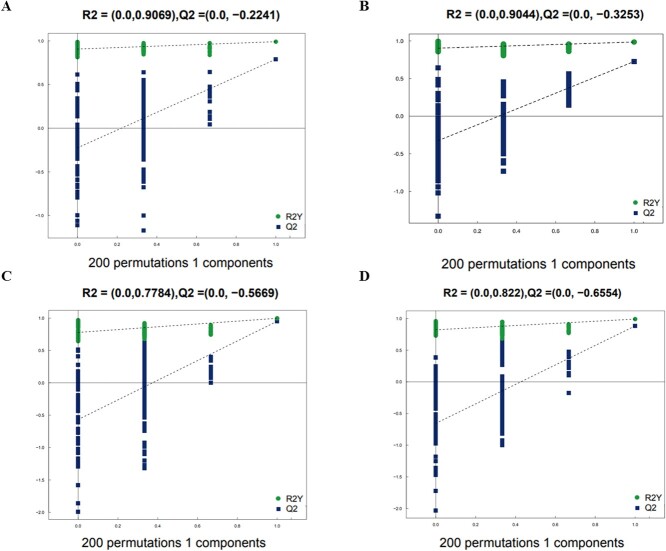
Permutation test (base on 200 permutations) of urine metabolites between NM group and AI group in captive male and female giant pandas. R^2^Y indicated the model’s explanation for the Y variable rate; Q^2^ indicated the predictive ability of the model.

Based on univariate statistical analysis, we conducted a comprehensive examination of the urine metabolomics data from captive adult male and female giant pandas exhibiting different NM behaviours (normal and abnormal). We analysed all detected metabolites (FC > 1.5 or FC < 0.67, *P* < 0.05) in both positive and negative ion modes, visualizing the results through volcanic maps. Subsequently, we identified the top 10 upregulated and downregulated metabolites with qualitative names for labelling (Fig. 2). Furthermore, we utilized multidimensional statistical analysis and Partial Least Squares Discrimination Analysis (OPLS-DA) to establish a model expressing the relationship between sample categories based on metabolite expression levels. The OPLS-DA model parameters of captive adult male giant pandas were obtained through 7-fold cross-validation as R^2^X = 0.313, R^2^Y = 0.991, Q^2^ = 0.79 in the positive ion mode and R^2^X = 0.372, R^2^Y = 0.986, Q^2^ = 0.727 in the negative ion mode. The model parameters for captive adult female giant pandas in negative ion mode were R^2^X = 0.478, R^2^Y = 0.991, Q^2^ = 0.883, whilst those in positive ion mode were R^2^X = 0.562, R^2^Y = 0.996, Q^2^ = 0.95. The results indicate good stability of the model (Fig. 3). To prevent overfitting during modelling, a permutation test was conducted to validate the model’s robustness and validity. The gradual decrease in permutation retention led to a corresponding decrease in R2 and Q2 of the random model, demonstrating that the original model did not suffer from overfitting and remained robust throughout the analysis process (Fig. 4).

#### Differential metabolites and bioinformatics analysis

**Figure 5:**
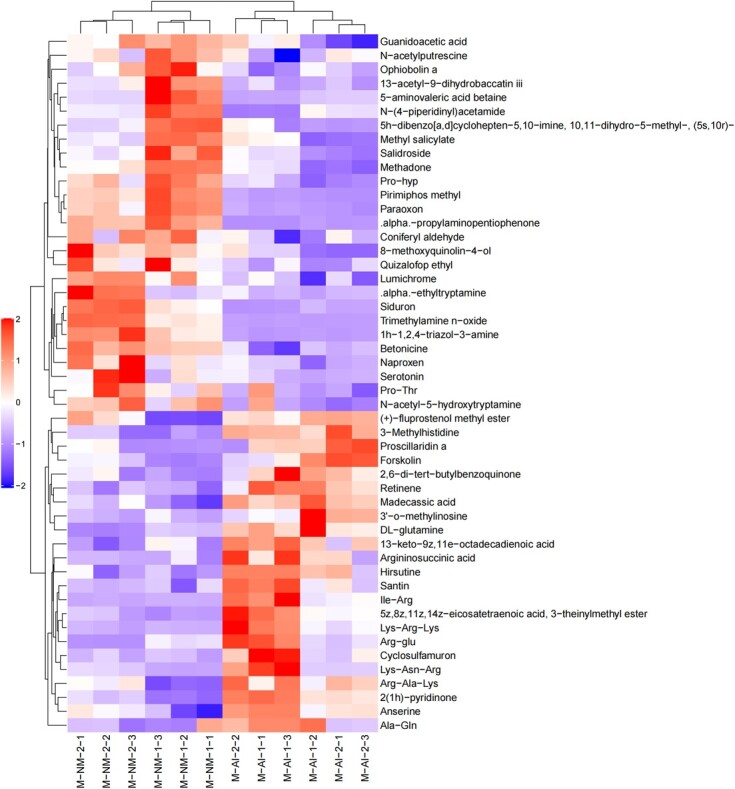
Bioinformatics analysis of differential metabolites between NM group and AI group in captive male giant pandas: significant differential metabolite hierarchical clustering results of male giant pandas in captivity under positive ion mode. Note: Each row in the figure represents a differential metabolite, with the ordinate indicating significantly differentially expressed metabolites, and each column representing a group of samples, with the abscissa providing sample information. Metabolites exhibiting similar expression patterns are clustered together on the left.

**Figure 6:**
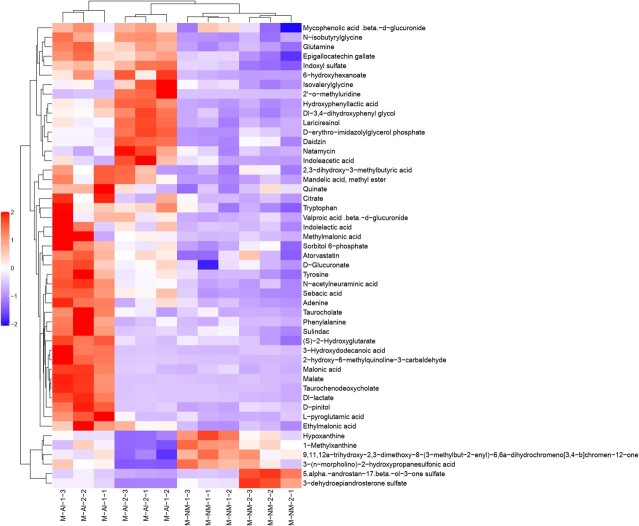
Bioinformatics analysis of differential metabolites between NM group and AI group in captive male giant pandas: significant differential metabolite hierarchical clustering results of male giant pandas in captivity under negative ion model.

**Figure 7:**
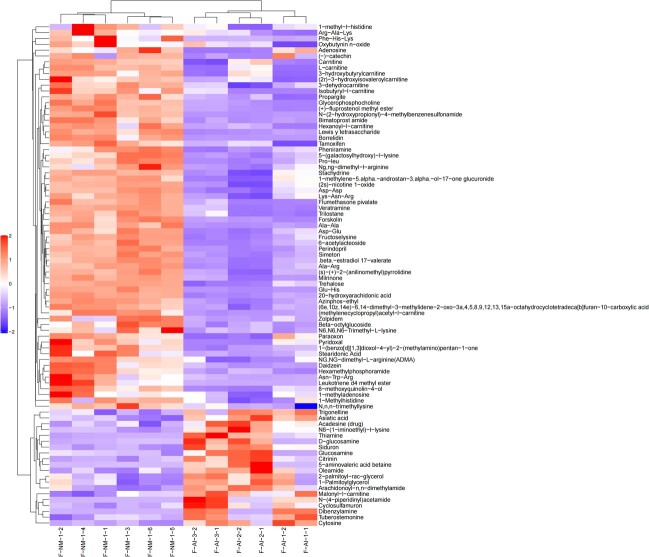
Bioinformatics analysis of differential metabolites between NM group and AI group in captive female giant pandas: significant differential metabolite hierarchical clustering results of female giant pandas in captivity under positive ion model.

**Figure 8:**
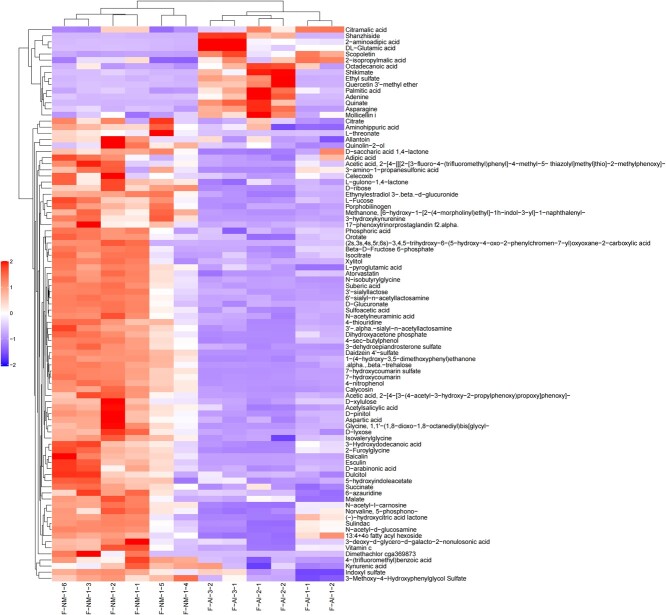
Bioinformatics analysis of differential metabolites between NM group and AI group in captive female giant pandas: Significant differential metabolite hierarchical clustering results of female giant pandas in captivity under negative ion model.

In this experiment, strict OPLS-DA VIP > 1 and *P-*value <0.05 were utilized as the criteria for significant difference metabolite screening. A total of 94 different metabolites was identified in the urine of adult male giant pandas in captivity, with 46 metabolites detected in the positive mode (Fig. 5) and 48 in the negative mode (Fig. 6). When compared to the M-NM group, a decrease was observed in 74 urine metabolites such as malic acid, citrate, phenylalanine, glutamine, tryptophan and tyrosine in giant pandas from the M-AI group; conversely, an increase was noted in 20 urine metabolites including serotonin, 5-aminovalerate and betaine. A total of 177 different metabolites was identified in the urine of adult female giant pandas in captivity, with 86 metabolites detected in positive mode (Fig. 7) and 91 metabolites in negative mode (Fig. 8). In comparison to the F-NM group, the F-AI group exhibited a decrease in 35 urine metabolites such as malic acid, succinic acid, DL-glutamic acid and aspartic acid, whilst an increase was observed in 144 urine metabolites such as kynurenic acid and 5-aminovaleric acid betaine.

**Figure 9:**
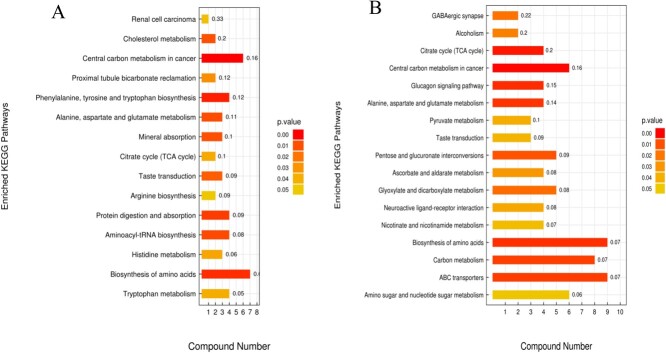
KEGG pathway enrichment analysis of differentially expressed metabolites between NM group and AI group in captive male and female giant pandas; A: male giant pandas in captivity; B: female giant pandas in captivity. Note: The vertical axis of the bar graph represents each KEGG metabolic pathway, whilst the horizontal axis denotes the number of differentially expressed metabolites within each pathway. The colour gradient reflects the *P*-value of the enrichment analysis, with darker shades indicating smaller *P*-values and greater significance in enrichment. Additionally, the numerical values on each column represent the rich factor.

Metabolic pathway enrichment analysis using KEGG was conducted on urine metabolites from captive adult male and female giant pandas. The results indicated that the expression of NM behaviour in captive adult male giant pandas is associated with changes in the metabolic pathways of phenylalanine, tyrosine and tryptophan biosynthesis, biosynthesis amino acid, propionate metabolism, protein digestion and absorption, as well as tryptophan metabolism (Fig. 9A). Additionally, the expression of NM behaviour in captive adult female giant pandas was associated with changes of the metabolic pathways primarily encompassing the citrate cycle (TCA cycle), biosynthesis amino acid, alanine, aspartate and glutamate metabolism, and γ-aminobutyric acid synaptic formation pathway (Fig. 9B).

#### Differences of cortisol and epinephrine in urine of giant pandas with different mating behaviours

There were no significant differences in cortisol and epinephrine levels between the two groups (cortisol: *P* = 0.746; epinephrine: *P* = 0.179) (Table 4).

**Table 4:** Urine cortisol and epinephrine levels of captive adult pandas

	Cortisol/(ng·mg^−1^)	Epinephrine/(ng·mg^−1^)
NM	24.716 ± 12.177	4.560 ± 2.310
AI	26.273 ± 12.871	5.934 ± 1.769
*P*	0.746	0.179

#### Differences in cortisol and epinephrine in the urine of giant pandas of different sexes with different mating behaviours

The results of urine cortisol revealed that female giant pandas in the NM group exhibited significantly lower levels of urine cortisol compared to those in the AI group (*P* = 0.006). Conversely, male giant pandas in the NM group showed higher levels of urine cortisol compared to those in the AI group, with a trend towards approaching significance (*P* = 0.088). Additionally, among all giant pandas displaying NM behaviour, male individuals demonstrated significantly higher urine cortisol content than their female counterparts (*P* = 0.033), whereas male giant pandas unable to exhibit NM behaviours displayed significantly lower urine cortisol content than females (*P* = 0.026) (Table 5).

**Table 5:** Urine cortisol and epinephrine levels of captive adult pandas

	Cortisol/(ng·mg^−1^)		*P*-value	Epinephrine/(ng·mg^−1^)		*P*-value
	Female	Male		Female	Male	
NM	18.592 ± 4.159	33.844 ± 16.167	0.033	4.584 ± 1.437	4.540 ± 3.189	0.978
AI	37.474 ± 16.788	18.764 ± 6.207	0.026	6.693 ± 2.022	4.669 ± 0.713	0.155
*P*-value	0.006	0.088		0.094	0.948	

The results of urine epinephrine analysis indicated that the levels in the female NM group were lower than those in the female AI group, with a trend towards significance (*P* = 0.094). However, no significant differences were observed between the male NM group and the male AI group (*P* = 0.948).

## Discussion

The factors affecting the natural breeding efficiency of captive giant pandas are complex and dynamic (Swaisgood *et al.,* 2004; Zhang *et al.,* 2004; Li *et al.,* 2017). Given the conspicuous mate selection behaviour documented in free-ranging giant pandas, the academic consensus suggests that the diminished reproductive success in captivity may be attributed to the absence of autonomous mate choice, which could lead to enforced copulation with mismatched partners and consequent psychological stress (frustration) (Zhang *et al.,* 2004; Martin-Wintle *et al.,* 2015). However, this speculation lacks direct evidence or markers. Currently, our understanding of the adaptive state of captive giant pandas remains limited when relying solely on behavioural and hormonal manifestations. This metabolic response to stress represents a physiological reaction of the organism to external stimuli, leading to perturbations in internal homeostasis and subsequent modifications in metabolic pathways. In our investigation, we employed UPLC-Q-TOF/MS metabolomics technology to analyse urine samples from captive adult giant pandas to explore the hypothesis that the manifestation of NM behaviour in captivity is associated with psychological distress arising from incompatible pairings.

The results indicated significant differences in urine metabolite abundance between the NM and AI groups in both adult male and female giant pandas. The discrepancies observed may be correlated with substantial alterations in amino acid metabolic pathways, encompassing the activation of pathways tied to psychological status, such as those involving tryptophan, alanine, aspartate and glutamate metabolism. Previous research has shown that disruptions in tryptophan metabolic pathways could potentially result in imbalances of active metabolites, which are closely associated with psychiatric disorders such as depression and schizophrenia (Alkhalaf and Ryan 2015). The study also revealed a significant positive correlation between pathways related to amino acids such as alanine, aspartic acid and glutamate metabolism with outcomes of successful mating attempts. Consistent with these findings, our study observed a significant upregulation of metabolites (aspartic acid, DL-glutamic acid and citrate) associated with the metabolic pathways of alanine, aspartic acid and glutamate in the urine of captive adult giant pandas exhibiting normal NM behaviour. These results suggested that the expression of NM behaviour in captive giant pandas is linked to their emotional state.

Our study revealed that the urinary serotonin levels in male pandas from the AI group were lower compared to those in the NM group, and there were significant alterations in the metabolites associated with tryptophan metabolism. Additionally, in the AI group, there was a notable enrichment in metabolic pathways related to the nervous system (GABAergic synapses), along with significant increases or decreases in inhibitory neurotransmitter amino acids and metabolites linked to brain development (DL-glutamic acid, succinic acid and 5-aminovalerate betaine). These changes in metabolites are considered potential risk markers for stress-related disorders (Deakin, 1996; Leppik *et al.,* 2018; Haikonen *et al.,* 2022), supporting the scientific hypothesis that psychological frustration due to pair incompatibility is a key factor contributing to the decline of NM behaviour among captive giant pandas.

By employing metabolomics techniques, we have initiated an investigation into the complex physiological changes occurring across all metabolites and metabolic pathways within the body. Our initial analysis has indicated that the abnormal expression of NM behaviours in captive giant pandas may be attributed to psychological stress resulting from incompatible pairings. To further support our hypothesis and gain deeper insights, we have also utilized ELISA methodologies to precisely quantify the levels of stress-related hormones (cortisol and epinephrine) during the mating season of these captive pandas. Our findings suggest that female giant pandas in the AI group exhibit significantly elevated levels of urinary cortisol compared to those in the NM group, indicating potential hindrance of NM behaviour due to frustration from pair incompatibility among adult female giant pandas. Additionally, previous research has demonstrated a positive association between urinary cortisol levels and stereotyped behaviour exhibited by giant pandas (Pan *et al.,* 2022). The most recent study has revealed that the stereotyped behaviour of giant pandas is associated with the success rate of NM. Specifically, male giant pandas exhibiting more stereotyped behaviours demonstrate a higher likelihood of successful NM during the mating season, potentially attributed to heightened sexual desire and fertility (Martin-Wintle *et al.,* 2020). Conversely, the stereotyped behaviour of female captive pandas exhibits a negative correlation with the expression of NM behaviour (Martin-Wintle *et al.,* 2020). This may be due to highly stereotyped female pandas inadvertently signalling a lack of sexual receptivity to male pandas, thereby diminishing their attractiveness (Mason, 2010). Our results on urinary cortisol levels in the urine of giant pandas also support the significance of the behavioural observations reported by Martin-Wintle *et al.* (2020). Specifically, our results indicate that male pandas exhibit more frequent stereotyped behaviours and are more likely to mate naturally when their urine cortisol levels are higher, whereas female pandas show the opposite pattern, with lower urine cortisol levels being associated with greater ease of NM. While there were no statistically significant differences in the epinephrine indicators pointing to chronic stress, a trend towards chronic stress was observed. Numerous human clinical studies have established a link between chronic psychological stress and dysfunction of the HPAA. Modulating the activity of the HPAA impacts the function of the sympathetic–adrenal–medullary system, leading to the release of epinephrine (Schmalbach *et al.,* 2020), influencing the human autonomic nervous system and eliciting physiological and behavioural manifestations of psychological disorders (Olff, 1999). Due to the lack of clear indicators for assessing chronic stress in animals and psychological stress resulting from chronic stress, particularly in studies involving wild animals, we must rely on relevant research findings from human studies for guidance. Additionally, based on preliminary urine metabolomics results, there is evidence suggesting that neurotransmitter metabolites and pathways associated with psychological stress may be linked to the reproductive success of captive giant pandas (Zhang *et al.,* 2022). For the subsequent investigation, it is imperative to integrate the behavioural science of giant pandas. The correlation between the aberrant manifestation of NM behaviour and chronic stress in captive giant pandas was examined through the utilization of brain tissue physiology, immunology and neurobiology.

In summary, the findings of this study suggest that the unconventional display of NM behaviour in captive adult giant pandas may be linked to physiological distress caused by incompatible pairings within restricted habitats. These results offer a theoretical foundation for the scientific conservation of captive giant panda populations and the enhancement of their welfare.

## Data Availability

Data will be provided on request to the first author (X.W.).
